# Stochastic Resonance Controlled Upregulation of Internal Noise after Hearing Loss as a Putative Cause of Tinnitus-Related Neuronal Hyperactivity

**DOI:** 10.3389/fnins.2016.00597

**Published:** 2016-12-27

**Authors:** Patrick Krauss, Konstantin Tziridis, Claus Metzner, Achim Schilling, Ulrich Hoppe, Holger Schulze

**Affiliations:** ^1^Experimental Otolaryngology, ENT-Hospital, Head and Neck Surgery, Friedrich-Alexander University Erlangen-NürnbergErlangen, Germany; ^2^Biophysics Group, Department of Physics, Center for Medical Physics and Technology, Friedrich-Alexander University Erlangen-NürnbergErlangen, Germany; ^3^Department of Audiology, ENT-Hospital, Head and Neck Surgery, Friedrich-Alexander University Erlangen-NürnbergErlangen, Germany

**Keywords:** dorsal cochlear nucleus, Zwicker tone, computational model, auditory nerve

## Abstract

Subjective tinnitus is generally assumed to be a consequence of hearing loss. In animal studies it has been demonstrated that acoustic trauma induced cochlear damage can lead to behavioral signs of tinnitus. In addition it was shown that noise trauma may lead to deafferentation of cochlear inner hair cells (IHC) even in the absence of elevated hearing thresholds, and it seems conceivable that such hidden hearing loss may be sufficient to cause tinnitus. Numerous studies have indicated that tinnitus is correlated with pathologically increased spontaneous firing rates and hyperactivity of neurons along the auditory pathway. It has been proposed that this hyperactivity is the consequence of a mechanism aiming to compensate for reduced input to the auditory system by increasing central neuronal gain, a mechanism referred to as homeostatic plasticity (HP), thereby maintaining mean firing rates over longer timescales for stabilization of neuronal processing. Here we propose an alternative, new interpretation of tinnitus-related development of neuronal hyperactivity in terms of information theory. In particular, we suggest that stochastic resonance (SR) plays a key role in both short- and long-term plasticity within the auditory system and that SR is the primary cause of neuronal hyperactivity and tinnitus. We argue that following hearing loss, SR serves to lift signals above the increased neuronal thresholds, thereby partly compensating for the hearing loss. In our model, the increased amount of internal noise—which is crucial for SR to work—corresponds to neuronal hyperactivity which subsequently causes neuronal plasticity along the auditory pathway and finally may lead to the development of a phantom percept, i.e., subjective tinnitus. We demonstrate the plausibility of our hypothesis using a computational model and provide exemplary findings in human patients that are consistent with that model. Finally we discuss the observed asymmetry in human tinnitus pitch distribution as a consequence of asymmetry of the distribution of auditory nerve type I fibers along the cochlea in the context of our model.

## Introduction

In western civilizations, 10–15% of the general population suffer from subjective tinnitus (Heller, 2003), the perception of a sound in the absence of any acoustic stimulus. In severe cases this phantom percept may lead to comorbidities like insomnia or psychological disorders like depression resulting in the inability to work or even suicide (Coles, 1984; Lewis et al., 1994; Langguth et al., 2011). Tinnitus is often accompanied by a hearing loss (Heller, 2003) and recent animal studies indicate that even relatively mild acoustic traumata which do not result in permanent elevation of hearing thresholds may lead to a massive loss of inner hair cell (IHC) synapses (synaptopathy) causing a so called hidden hearing loss (Liberman et al., 2015).

Despite the high prevalence and distress of affected patients, an effective cure for tinnitus still does not exist, since the exact mechanisms within the auditory system leading to the development of tinnitus are still unknown, and current models for tinnitus development are a matter of controversial debate (Gerken, 1996; Eggermont, 2003; Eggermont and Roberts, 2004; Engineer et al., 2011; Knipper et al., 2011; Schaette and McAlpine, 2011; Rüttiger et al., 2013). One class of such models proposes a mechanism within the central auditory system called homeostatic plasticity (HP) to be causative for tinnitus development. There, prolonged changes in the mean firing rates of the auditory nerve (AN), e.g., reduced AN activity caused by acoustic trauma are assumed to cause HP to readjust these mean firing rates to pre-trauma levels by means of increased neuronal gain, thereby compensating for the reduced cochlear input. This increased neuronal gain which is considered to emerge within long time scales (days to weeks) then leads to increased spontaneous firing rates within the auditory system which are hypothesized to be the correlate of the maladaptive hyperactivity observed in tinnitus (Knipper et al., 2011; Schaette and McAlpine, 2011). In support of current HP models it was argued that changes in firing rate, e.g., after a hearing loss event, may be detected by calcium-depended sensors that then regulate glutamate receptor trafficking (Turrigiano, 2008), thereby stabilization firing rates. This stabilization in turn is considered to be beneficial in terms of neuronal processing within these long time scales (Brenner et al., 2000; Dragoi et al., 2000; Schwartz and Simoncelli, 2001; Simoncelli and Olshausen, 2001; Dean et al., 2005, 2008; Dunn and Rieke, 2006; Robinson and McAlpine, 2009) but such a mechanism would not be helpful on short time scales for the detection of signals below the new threshold.

**Figure 1:**
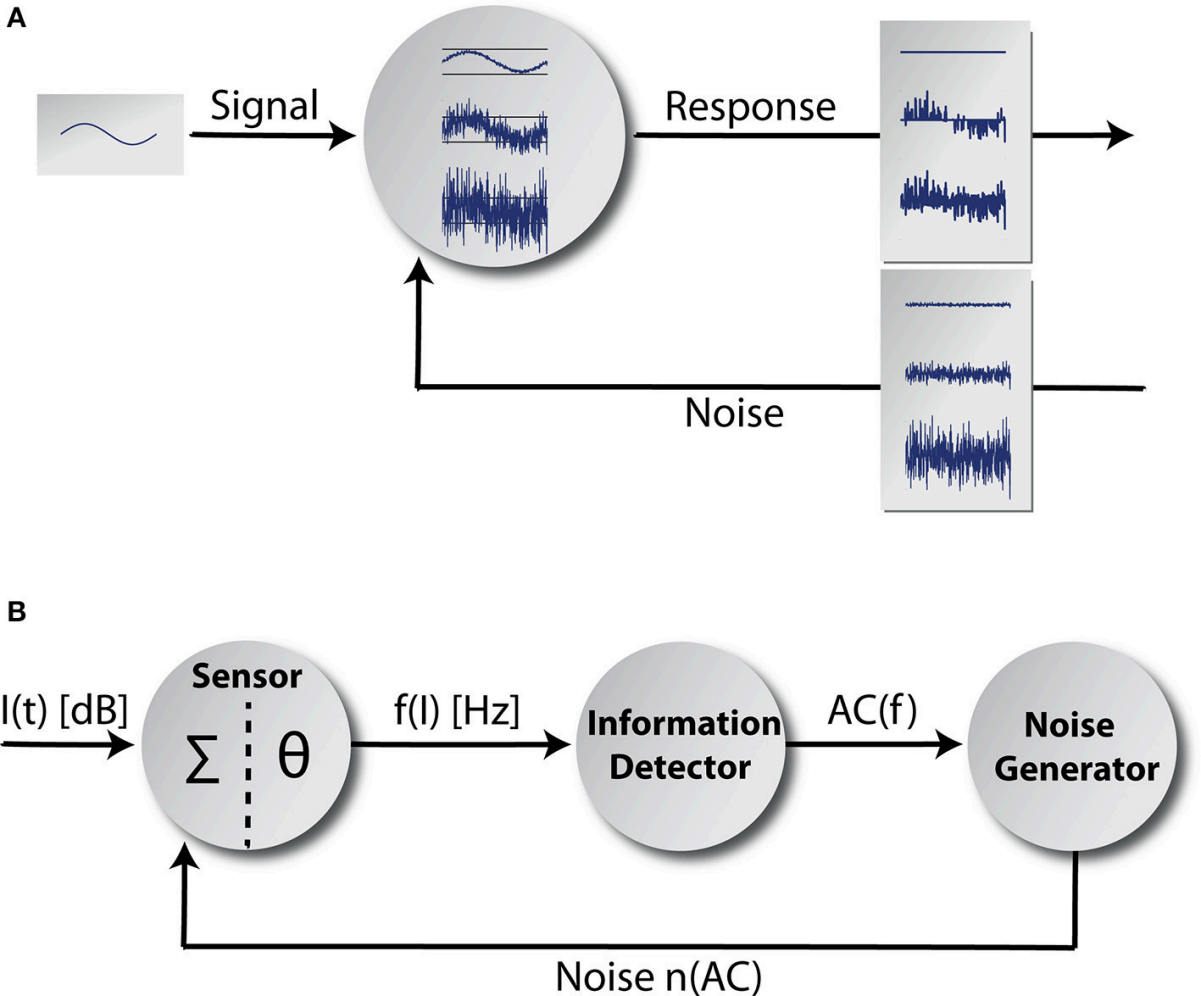
**Adaptive stochastic resonance**. In **(A)** a sketch of the adaptive SR principle is shown. Some kind of sub-threshold signal serves as input to a detector. The detector response is shown for three different levels of noise intensity. If the amount of noise is too small, the signal cannot be transmitted and if the amount of noise is too large the signal vanishes within noise. In contrast for the optimal level of noise, signal transmission is best. The core model is shown in **(B)**. It mainly consists of three functional units and a feedback-loop. The information detector calculates the autocorrelation of the time course of AN firing rate *f(I)* originating at a sensor (with a summation function Σ and a threshold θ*;* Equation 7) reflecting its information content. The noise generator is controlled by the information detector and feeds noise back to the sensor via feedback connections (for details refer to the Discussion section).

Here, inspired by information theory (Shannon, 1948), we propose an alternative and entirely new interpretation of tinnitus-related development of neuronal hyperactivity based on stochastic resonance (SR). SR refers to the phenomenon that weak signals that are sub-threshold for a given sensor (or synapse) still can be detected and transmitted by that sensor if noise is added to the sensor input (Figure 1A; Benzi et al., 1981; Collins et al., 1996; Levin and Miller, 1996; Gammaitoni et al., 1998). In that sense we follow the idea that any signal first has to be detected by a sensor before it could be amplified by increased neuronal gain. Or to use an analogy if the radio reception is bad due to a broken antenna it does not help to turn up the volume. Thus, the two main differences between our SR model and the HP models are, firstly that SR works prior of the detection threshold while HP influences processing after the detection threshold, and secondly the SR model assigns a functional role to spontaneous activity in the auditory system in contrast to the HP and other models, where increased spontaneous firing rates are simply a side-effect of the brain's attempt to cope with hearing loss.

SR has been found ubiquitously in nature covering a wide range of systems in physical and biological contexts (Wiesenfeld and Moss, 1995) and especially within the context of neuroscience (Douglass et al., 1993; Faisal et al., 2008; Mino, 2014). In addition, the existence of an optimal, non-zero intensity for the added noise has been demonstrated, allowing maximization of information transmission (Wiesenfeld and Moss, 1995). In self-adaptive signal detection systems based on SR, the optimum noise level is continuously adjusted via a feed-back loop, so that the system response in terms of information throughput remains optimal, even if the properties of the input signal change. For this processing principle the term adaptive SR has been coined (Mitaim and Kosko, 1998, 2004; Wenning and Obermayer, 2003). An objective function to quantify information content is the mutual information between the sensor input and output (Shannon, 1948). In the context of SR the mutual information is frequently used in theoretical approaches (Levin and Miller, 1996; Mitaim and Kosko, 2004; Moss et al., 2004). The choice of the mutual information is natural since the fundamental purpose of any transducer is to transmit information into a subsequent information processing system. It has been shown previously that the mutual information as a function of noise intensity has a maximum that indicates the optimal level of noise to be added to the input signal to achieve optimal information transmission by SR (Moss et al., 2004). However, a fundamental drawback of the mutual information is the impossibility of calculating it in any application of adaptive SR where the signal to be detected is unknown (Krauss et al., 2015). Furthermore, even if the underlying signal is known, the use of the mutual information still seems to be rather impractical within the context of neural network architectures, since calculating the mutual information requires evaluation of probability distributions, logarithms, products and fractions, i.e., operations that are hard to implement in neuronal networks. In a previous work (Krauss et al., 2015) we were able to show that this fundamental drawback can be overcome by another objective function, namely the autocorrelation of the sensor response. There we introduced the concept of the success probability and proved analytically and numerically that firstly, as a function of noise intensity, this quantity has a well-defined peak indicating the optimal level of noise for SR and secondly that mutual information and autocorrelation can be expressed as strictly monotonous functions of the success probability. Hence both, mutual information and autocorrelation, exhibit their maximum at the same level of noise and consequently, maximizing the output autocorrelation leads to similar or even identical estimates of optimal noise intensities for SR as the mutual information, yet with the decisive advantage that no knowledge of the input signal is required (Krauss et al., 2015). In contrast to the mutual information, the evaluation of autocorrelation functions may easily be implemented within neuronal networks using delay-lines and coincidence detectors (Licklider, 1951).

Taken together, we here propose that adaptive SR based on maximizing the output autocorrelation is a major adaptive principle in the auditory system that operates both on short and long time scales to maintain optimal information transmission in cases of changing statistics of the IHC output (= input to the auditory system), e.g., due to cochlear damage. In case of such reduced IHC output, first, the target neurons' autocorrelation decreases. As a consequence, second, the internal noise generated within the auditory system is increased and fed back to the sensor level to compensate for lost information transmission by means of SR. By that, this mechanism is able to at least partially restore hearing thresholds which have been elevated due to noise trauma. We here propose that in case of chronic cochlear damage this adaptively changed internal noise is increased permanently and hence a possible correlate of the hyperactivity often associated with subjective tinnitus. Note that in order to demonstrate the basic concept and principles of adaptive SR in the context of cochlear acoustic traumata and tinnitus development we here present a single frequency channel model approach and focus on within-channel mechanisms only. Putative cross-talk between neighboring frequency channels will be addressed in a follow up study. Furthermore, and in line with our model, we demonstrate in a cohort of over 39,000 patients that those with tinnitus have significantly improved hearing thresholds in the low frequency range compared to those without tinnitus. We discuss the possibility that the asymmetric distributions of the different type I AN fibers along the cochlea in combination with our SR model may explain the overrepresentation of high pitched tinnitus found in patients.

## Methods

We implemented a phenomenological single frequency channel model (Figure 1B) comprising the acoustic stimuli (function *I(t)* of the sound intensity levels), the first synapse from the IHC (cf. Discussion) to the AN (sensor with a summation function ∑ and a threshold θ), the AN responses *f(I)* and the effects of cochlear damage to AN responses. Furthermore, the model includes the adaptive SR principle based on the mean autocorrelation of the AN responses *AC(f)* calculated at the information detector whose output modulates the activity of a noise generator that feeds the noise *n(AC)* back into the sensor. We model coarse-grained functional units: to simplify matters, we focused on input-output mappings rather than on single neuron models or concrete neural network architectures. Nevertheless, we emphasize that each part of the adaptive SR feedback-loop is highly biologically plausible and may be implemented as a neuronal network in a more fine-grained model. Possible candidate structures within the auditory system where the functional units of our model could be realized will be discussed in the Discussion section.

All parts of the model were implemented using the programming language C/C++.

### Distribution of sound intensities of acoustic stimuli

**Figure 2:**
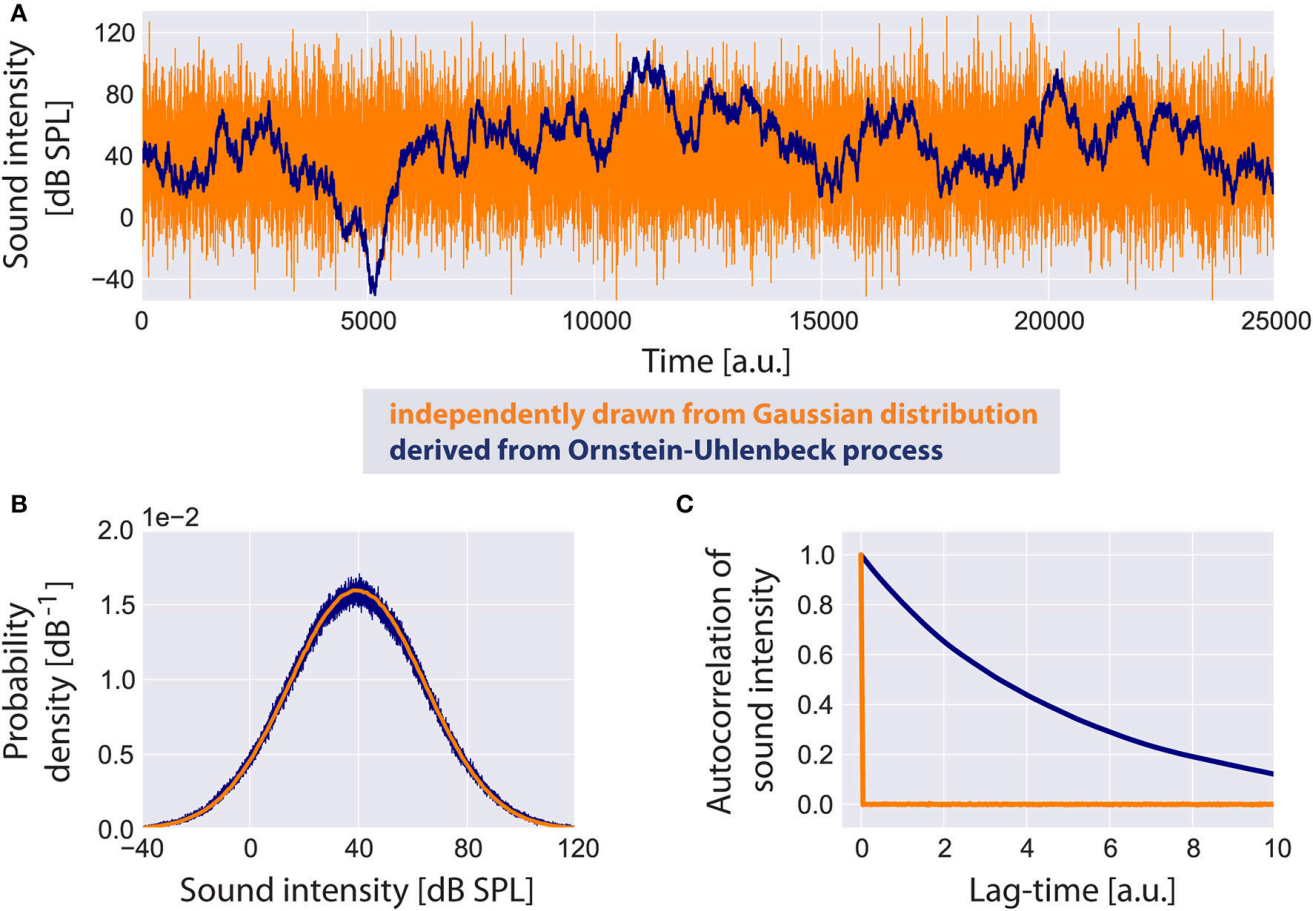
**Standard acoustic environment**. Two sample time series of sound intensities are shown **(A)**: the uncorrelated time series (orange) has been generated by independently drawing values from a Gaussian distribution, whereas the correlated time series serving as input to the model (blue) is derived from an Ornstein-Uhlenbeck process. Although both time series look very different, their probability density functions **(B)** are identical. The autocorrelation function of the uncorrelated time series has a peak at lag-time zero. The autocorrelation of the Ornstein-Uhlenbeck process decreases exponentially with increasing lag-time **(C)**.

Based on a computational model presented by Schaette and Kempter (2006) we assume the probability density function of the sound intensity levels *I* (in dB SPL) of the acoustic input (Figure 2A) to the model to be Gaussian (Figure 2B) with a mean value μ_*I*_ of 40 dB and standard deviation σ_*I*_ of 25 dB:

(1) $$p_{I}(I)=\frac{1}{\sqrt{2\pi \sigma_{I}^{2}}}\text{exp}\left(-\frac{{\left(I-\mu_{I}\right)}^{2}}{2\sigma_{I}^{2}}\right)$$

Note that this Gaussian distribution of sound intensity levels in dB corresponds to a log-normal distribution of the linear amplitudes of the sound stimuli. As input to the model we do not draw independent samples from this distribution during simulation. Instead, in order to generate an autocorrelated time series of sound intensities (Figure 2C), yet with identical mean value and standard deviation as in the uncorrelated case (Figure 2B), we implement an Ornstein-Uhlenbeck process (Uhlenbeck and Ornstein, 1930) that satisfies the stochastic differential equation:

(2) $$dI_{t}=\left(\mu_{I}-I_{t}\right)dt+\;\sqrt{2\sigma_{I}^{2}}\;dW_{t}$$

where *W*_*t*_ is the Wiener process (Einstein, 1905).

It is plausible to assume sound intensities to be autocorrelated especially for meaningful acoustic stimuli like speech or music. We refer to this random walk through sound intensities as the standard acoustic environment.

### Modeling of auditory nerve responses

The firing rate *f(I)* of the AN at a sound intensity *I(t)* is modeled analogous to Schaette and Kempter (2006) with a threshold *I*_θ_ of 0 dB SPL, spontaneous firing rate *f*_*sp*_ of 50 Hz and maximum firing rate *f*_*max*_ of 250 Hz. The response function *f(I)* is assumed to be adapted to the distribution of sound intensities. This means that for *I* > *I*_θ,_ *f(I)* is proportional to the normalized cumulative distribution function $\int_{I_{\theta }}^{I}p_{I}\left(I^{\prime }\right)dI^{\prime }$ of the sound intensities hence, according to the infomax principle, *f(I)* has maximum information on *I(t)* (Laughlin, 1981):

(3) $$f\left(I\right)=\{\begin{array}{ll} f_{sp} & for\;I<I_{\theta }\\ f_{sp}+\;\left(f_{max}-f_{sp}\right)\frac{\int_{I_{\theta }}^{I}p_{I}\left(I\prime \right)dI\prime }{1-P_{sp}} & for\;I\geq I_{\theta } \end{array}$$

with $P_{sp}=\;\overset{I_{\theta }}{\underset{I_{-\infty }}{\int }}p_{I}\left(I\right)dI$ the probability of occurrence of spontaneous activity.

**Figure 3:**
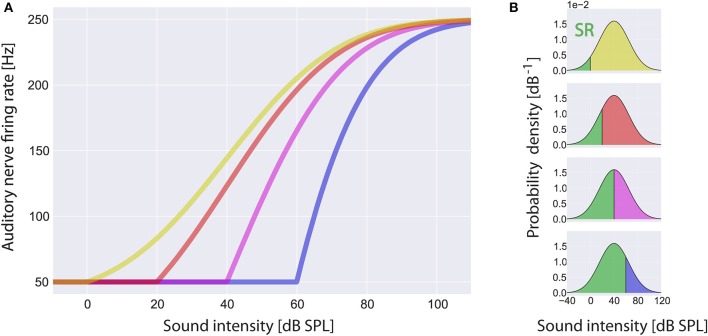
**Rate-intensity-functions used in the model**. Shown are sample rate-intensity-functions **(A)** for different thresholds *I*_θ_ at 0 dB SPL (red), 20 dB SPL (green), 40 dB SPL (yellow) and 60 dB SPL (blue). In each case the spontaneous firing rate *f*_*sp*_ is 50 Hz and the maximum firing rate *f*_*max*_ is 250 Hz. In **(B)** the ratios of sub- and supra-threshold sound intensities are illustrated for increasing thresholds from top to bottom. Note that stochastic resonance (SR) only takes place in the range of sub-threshold sound intensities (green areas).

Within the scope of this article we focus on changes of the threshold *I*_θ_ due to cochlear damage only and do not take into account changes of the spontaneous firing rate *f*_*sp*_ or the maximum firing rate *f*_*max*_. In Figure 3A some example rate-intensity functions that are used in our computational model are shown for different thresholds. Note that with increasing threshold the fraction of sub-threshold sound intensities, i.e., the fraction where SR is effective, does also increase (Figure 3B).

### Autocorrelation function, mean autocorrelation, and mutual information

The autocorrelation function of a time-dependent variable *x(t)* is defined as:

(4) $$AC\left(\tau \right)=\frac{\frac{1}{T}\sum_{t}\left(x\left(t\right)-\mu_{X}\right)\left(x\left(t+\tau \right)-\mu_{X}\right)}{\sigma_{X}^{2}}$$

where μ_*X*_ and σ_*X*_ are mean and standard deviation, respectively.

The mean autocorrelation is derived by averaging the autocorrelation function over all evaluated lag-times:

(5) $$AC=\;\frac{1}{N}\sum_{\tau =1}^{N}AC\left(\tau \right)$$

The mutual information of input *X* and output *Y* is defined as:

(6) $$MI\left(X;Y\right)=\;\int_{Y}\int_{X}p\left(x,y\right)\;log_{2}\left(\frac{p\left(x,y\right)}{p\left(x\right)p\left(y\right)}\right)dxdy$$

where *p*(*x*), and *p*(*y*) are the marginal probability density functions and *p*(*x, y*) is the joint probability density function of X and Y.

### Adaptive stochastic resonance model

**Figure 4:**
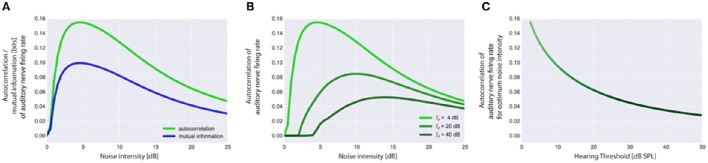
**Stochastic resonance and output autocorrelation. In (A)** typical resonance curves for the standard acoustic environment (cf. Methods) with hearing threshold *I*_θ_ = 4 dB are shown to illustrate the equivalence of the mean autocorrelation of the auditory nerve (AN) firing rates (output) and the mutual information between sound intensities (input) and AN firing rates (output). Both objective functions peak at the same noise intensity which is referred to as the optimum noise intensity. Resonance curves based on output autocorrelation are shown in **(B)** for the standard acoustic environment and three different hearing thresholds (4, 20, 40 dB). Note that with increasing hearing threshold the corresponding optimum noise intensity also increases whereas the maximum of output autocorrelation decreases **(B,C)**. Furthermore, the onset of the rise of the autocorrelation **(B)** is rightward shifted as the initial noise has to be increased more for larger values of the hearing threshold to produce effective SR.

**Figure 5:**
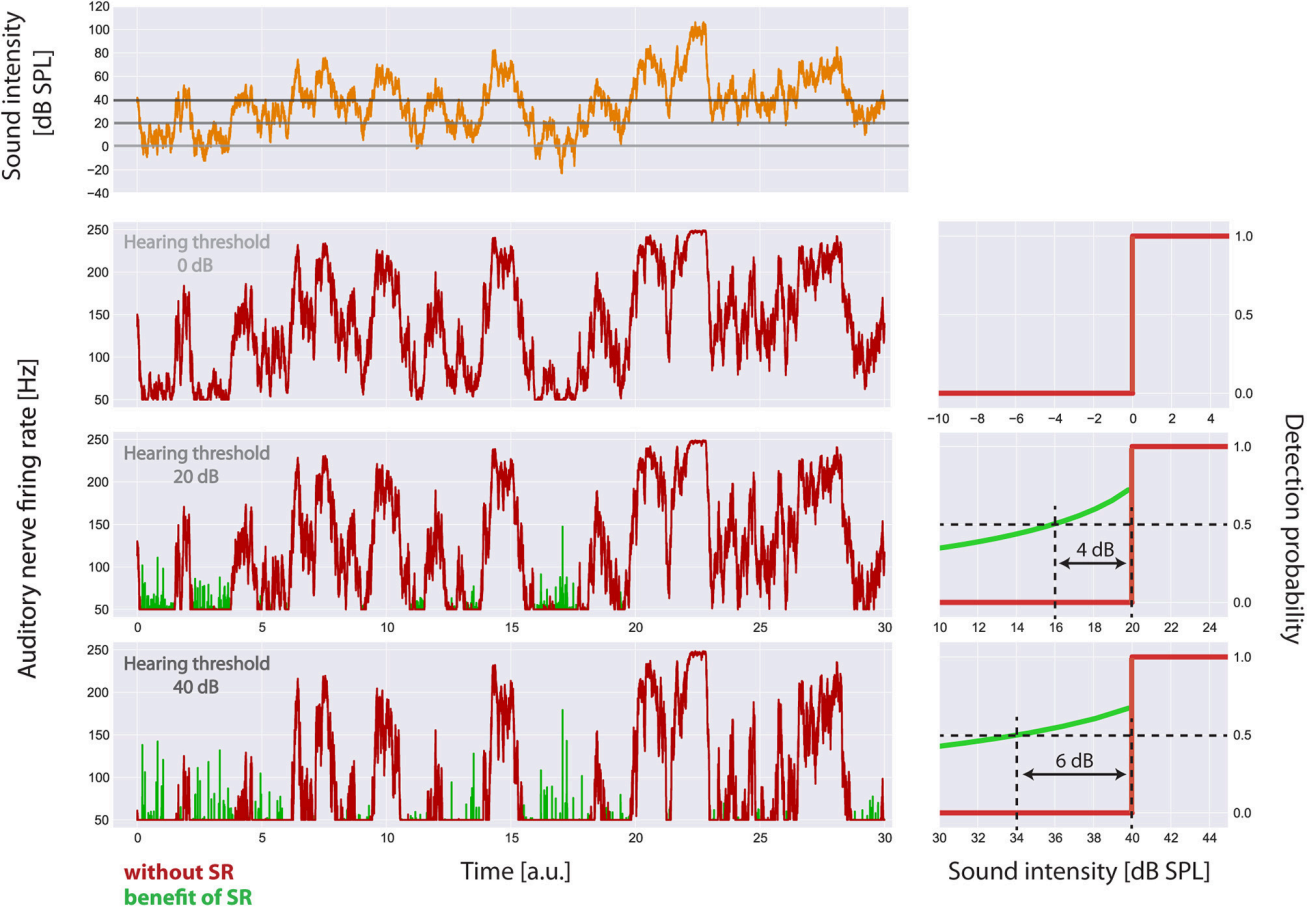
**Visualization of the main effects of the model**. For the standard acoustic environment (orange) the auditory nerve firing rate responses (left panels) and corresponding psychometric functions (detection probability as a function of sound intensity, right panels) are shown for different degrees of hearing loss (0, 20, 40 dB), both without SR (red) and with the optimum SR noise intensity (green). The hearing thresholds with the aid of SR are given by the sound level at 50% detection probability. Hence the benefit of SR is about 4 dB (at 20 dB hearing loss) or 6 dB (at 40 dB hearing loss), respectively.

**Figure 6:**
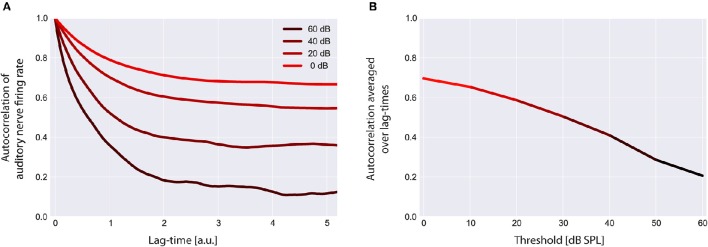
**Autocorrelation functions of the cochlear output**. Shown are the autocorrelation functions for different thresholds **(A)**. For increased thresholds the values of the autocorrelation function systematically shift to smaller values, reflecting the decreased amount of information content transmitted by the cochlea. The mean autocorrelation obtained by averaging the autocorrelation function over all evaluated lag-times decreases monotonically with increasing threshold **(B)**.

**Figure 7:**
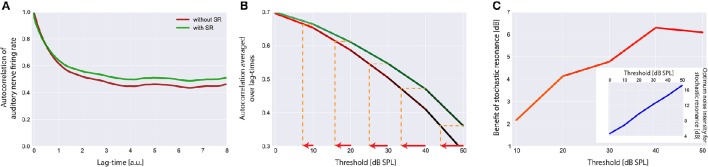
**Effect of stochastic resonance (SR)**. In **(A)** a sample autocorrelation function for a threshold *I*_θ_ of 30 dB is shown with (green) and without (red) the effect of SR. As can be clearly seen, SR is able to improve the autocorrelation. Note that an improvement of the autocorrelation is equivalent to improved hearing thresholds in our model. **(B)** Averaged autocorrelation as a function of the corresponding threshold. Horizontal iso-AC lines and arrows on the x-axis indicate the benefit of SR for different autocorrelation levels. **(C)** Benefit of SR on hearing threshold in dB for different threshold levels. Note that with increasing thresholds more noise is required to improve the autocorrelation (inset).

Our adaptive SR model mainly consists of three functional units and a feedback-loop (Figure 1B). The first unit, referred to as sensor (which we assume to be located within the cochlea, presumably at the post-synapse of the IHC, cf. Discussion), receives input from the environment, namely the time course of sound intensities *I(t)* which are generated using Equation (2). Its output, the AN firing rate *f(I)*, is calculated according to Equation (3). The second unit receives its input from the sensor and calculates the autocorrelation of the time course of AN firing rates *AC(f)* according to Equation (5), reflecting their information content. We refer to this unit as the information detector. The third unit is controlled by the information detector and injects white noise *n* with constant zero mean μ_*n*_ = 0 but tunable variance $\sigma_{n}^{2}$ back to the sensor via efferent connections. For this part of the system we use the term noise generator. We refer to $\sigma_{n}^{2}\;$ as the noise level. The model presumes that via feedback control the noise level for SR is set to a level where the autocorrelation function of the sensor output becomes maximal ($\sigma_{n,opt}^{2}\left(AC\right)$) which is equivalent to maximizing the mutual information (Figure 4A; cf. Krauss et al., 2015). The optimal noise level depends on both the signal amplitude and the threshold. All further simulations presented in this report (Figures 5–7) use these previously evaluated optimal noise levels $\sigma_{n,opt}^{2}\left(AC\right)$. Hence, in case of SR the sensor receives two kinds of inputs, namely the sound intensities *I(t)* and white noise *n(AC)* with variance $\sigma_{n,opt}^{2}\left(AC\right)$. Both inputs are summed (Σ) before thresholding (θ) occurs. Thus, equation (3) is slightly modified to:

(7) $$\begin{array}{l} \;\\ f\left(I+n(AC)\right)=\{\begin{array}{ll} f_{sp} & for\;I+n(AC)<I_{\theta }\\ f_{sp}+\;\left(f_{max}-f_{sp}\right)\frac{\int_{I_{\theta }}^{I\;+\;n(AC)}p_{I}\left(I\prime \right)dI\prime }{1-P_{sp}\;} & for\;I+n(AC)\geq I_{\theta } \end{array} \end{array}$$

The autocorrelation of the sensor output also does not only depend on the amount of injected noise $\sigma_{n,\;opt}^{2}\left(AC\right)$ but also on the hearing threshold, i.e., the larger the hearing threshold, the smaller is the autocorrelation at the optimal SR noise level (Figures 4B,C). Note that large hearing thresholds (Figure 4B) result in a rightward shift of the rising point of the autocorrelation as a function of noise intensity. As the noise intensity has to be higher to reach the threshold in the first place, the autocorrelation remains zero for lower noise intensities (Figure 4B). All resonance curves shown in Figure 4 were computed using the standard acoustic environment as input, yet with different thresholds (cf. figure caption).

### Patient data

Anonymized audiometric data from patients who came to the ENT clinic in Erlangen for medical examination were used. Therefore, no declaration of consent was required by German law. 78,282 data sets of pure tone audiometries of both ears were collected in 39,141 patients between the years 2000 to 2015. Patients were not characterized by their gender, age [median (25, 75% quantil): 42 (21, 58)] or by former or current pathologies not affecting hearing thresholds but only by their report of percieving a tinnitus (group T) or not (group NT). Standardized audiometric testing instruments of an audiological clinic were used. Air conduction thresholds were measured by pure-tone audiometry (stimuli were presented from −10 to 130 dB in 5 dB steps) for both ears separately for every patient. Test frequencies were 125, 250, 500, 750, 1000, 1500, 2000, 3000, 4000, 6000, and 8000 Hz. Hearing thresholds were tested pairwise for every frequency with a Kolmogorow Smirnoff test for two samples.

## Results

The aim of this study was to present an alternative to existing models of tinnitus-related development of neuronal hyperactivity. To this end, we will demonstrate how adaptive SR based on maximizing the autocorrelation function of the sensor (IHC synapse) output after chronically reduced input into the auditory system, e.g., due to cochlear damage, may lead to permanently increased internal noise within the auditory system (as a possible correlate of tinnitus). We first show the effect of increased thresholds on the autocorrelation function of the time series of AN firing rates *f(t)* and subsequently the effect of SR on the autocorrelation function.

### SR improves detection probability after hearing loss

In Figure 5 the main effects of the model on the auditory nerve firing rates are shown. For the standard acoustic environment (orange) the model's auditory nerve firing rate responses (left panels) and corresponding psychometric functions (detection probability as a function of sound intensity; right panels) are shown for different degrees of hearing loss (0, 20, 40 dB), both without SR (red) and with the optimum SR noise intensity (green). The hearing thresholds with the aid of SR are given by the sound level at 50% detection probability. As it turned out, the benefit of SR based on our model is about 4 dB (at 20 dB hearing loss) or 6 dB (at 40 db hearing loss), respectively.

### Reduced input decreases the mean autocorrelation function of the sensor output

Using our model we evaluated the autocorrelation function of the time series of simulated AN firing rates *f(I)* as defined in the Method section. In all simulations we used the standard acoustic environment as input to the sensor *I(t)*, namely a correlated random walk with normally distributed values with a mean value of 40 dB SPL and standard deviation of 25 dB (cf. Methods; Figure 2). In Figure 6A, autocorrelation functions for this input at different sensor thresholds are shown. For increased thresholds the values of the autocorrelation function systematically shift to smaller values. Accordingly, the mean autocorrelation obtained by averaging the autocorrelation function over all evaluated lag-times decreases monotonically with increasing threshold (Figure 6B). These data demonstrate, as could be expected, that the amount of information content transmitted by the sensor decreases with increasing sensor thresholds, that is, with increasing hearing thresholds.

### Stochastic resonance improves mean autocorrelation and increases internal noise after hearing loss

The effect of SR at the level of the sensor on the autocorrelation of the sensor output and its effect on hearing threshold is shown in Figure 7. Again the standard acoustic environment served as input. In Figure 7A sample autocorrelation functions for an exemplary threshold *I*_θ_ of 30 dB are shown with (green) and without (red) the effect of SR. Obviously, SR is able to increase the autocorrelation, that is, to improve information transmission. When averaging the autocorrelation over all lag times and plotting both functions (with and without SR) as a function of the threshold (Figure 7B) the benefit of SR on hearing threshold becomes obvious (arrows indicate threshold shift). In Figure 7C this benefit of SR in dB is plotted as a function of hearing loss, revealing maximal benefits of up to 6 dB.

Remarkably, the benefit of SR (i.e., improvement of hearing thresholds) according to both, detection probability (Figure 5) and improvement of autocorrelation (Figure 7) exhibit nearly identical values (4 or 6 dB, respectively).

This improvement of hearing thresholds leads to a readjustment of the rate-intensity function *f(I)* equivalent to a leftward shift of the onset of the rise of the *f(I)* in Figure 3A. On the other hand, with increasing thresholds more noise is required to optimally improve the autocorrelation (Figure 7C, inset). We here propose that in case of chronically elevated thresholds, e.g., due to cochlear damage, this internal noise added for SR to compensate for the elevated thresholds has to be increased permanently and hence may be the correlate of tinnitus-related neuronal hyperactivity. If this would be the case then tinnitus could be viewed as a side-effect of a compensatory mechanism of the auditory system that aims to (at least partly) restore hearing thresholds after hearing loss by adding internal noise to the sensor level, thereby making use of SR.

### Audiometric patient data in support of our hypothesis

**Figure 8:**
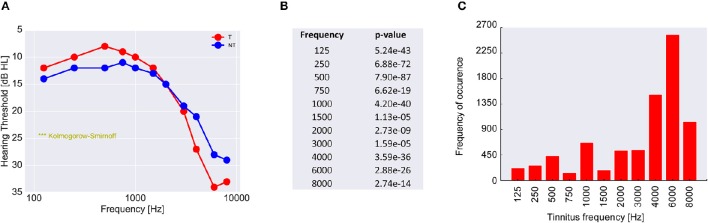
**Analysis of 39,141 patients with increased hearing thresholds (A)**. All patients are divided into two groups, namely those with (red) and those without (blue) tinnitus. Shown are the median hearing thresholds for each frequency. For lower frequencies the tinnitus patients have lower thresholds than the non-tinnitus patients, which is perfectly consistent with the prediction of our model. However, for higher frequencies tinnitus patients have higher hearing levels than non-tinnitus patients. Since the pitch of the perceived tinnitus mainly lies within the high-frequency region **(C)**, the tinnitus percept could cause a masking effect leading to secondarily increased hearing levels. In **(B)** the *p*-values of the statistical comparison of the data in **(A)** (two sample Kolmogorov-Smirnoff test) are summarized. For each tested frequency the distributions of hearing thresholds are significantly different for the two patient groups. Note that distributions still may be significantly different even if median values happen to be identical (as is the case at 3 kHz).

Finally, to scrutinize this hypothesis we analyzed the audiometric data of 39,141 patients from the ENT hospital Erlangen (Figure 8A). As it turned out and in line with our hypothesis, (tonal) tinnitus patients on average indeed had significantly better hearing thresholds than the non-tinnitus patients, namely in the frequency range below 3 kHz., i.e., the frequency range most important for speech processing (Figure 8B). This finding—at least for frequencies below 3 kHz—is consistent with the work of König et al. (2006) reporting better hearing thresholds for tinnitus patients compared to non-tinnitus patients in a range from 0.125 to 8 kHz. On the other hand, the majority of the patients (65.5%) reported tinnitus frequencies above 3 kHz (Figure 8C), i.e., in the frequency range where tinnitus patients on average had significantly higher hearing thresholds compared to the non-tinnitus patients.

## Discussion

In this report we have demonstrated how an information processing system based on adaptive SR aiming to maximize the autocorrelation of a sensor's output may cause neuronal hyperactivity in the case of chronically reduced input to that sensor. In this context we view this neuronal hyperactivity as a side effect of the adaptive SR mechanism whose main purpose is to optimize information transmission and thereby partly restore lost sensitivity by improving thresholds in cases of sensor damage.

### Plausibility of the model in the context of the anatomy of the auditory system

After phenomenologically describing the main concepts and mechanisms of our model we will now discuss how plausible the model may be in the context of the known anatomy of the auditory system. To this end we will try to identify candidate structures within the auditory system for the implementation of the model components as given in Figure 1B.

One candidate structure for the noise adding feedback loop predicted by our model is the efferent projection from the superior olivary complex to the IHC, the lateral olivocochlear bundle. For SR to be effective the internal noise fed back to the sensor must reach the post-synaptic site. Interestingly (and in contrast to efferent projections to the outer hair cells which directly contact the basolateral region of these cells via axosomatic synapses), the efferent projections from the superior olivary complex [especially the lateral olivocochlear bundle, a heterogeneous population of neurons utilizing several different neurotransmitter systems like acetylcholine, gamma-aminobutyric acid (GABA), glycine and dopamine Ruel et al., 2006, 2007; Dlugaiczyk et al., 2008] to the IHC form axodendritic synapses with the AN below the IHC (Groff and Liberman, 2003; Darrow et al., 2007) where they could modulate the response probability to sensory input by inhibition and excitation for SR, exactly at the site predicted by our SR model. The olivocochlear efferents are therefore able to modulate the AN post-synaptic membrane potential or—in terms of SR—feed noise into the sensor.

This prediction is in line with electrophysiological data from animal models (Dallos and Harris, 1978; Liberman and Kiang, 1978; Liberman and Dodds, 1984) where spontaneous rates of AN fibers after cochlear damage were either reduced or unchanged but never increased. In our model, the spontaneous rate would be initially decreased and then raised back to normal levels by the SR feedback.

Nevertheless, a recent study showed that most synaptic events were sufficient to trigger an action potential in spiral ganglion neurons (Rutherford et al., 2012). If they are responding to almost every bit of signal from the hair cell, it seems unlikely that noise injection into the spiral ganglion neurons could make them even more sensitive. Furthermore, it has been shown that one of the major functions of the lateral olivocochlear bundle feedback seems to be the regulation of responses to high sound intensities (Le Prell et al., 2003a), cochlear neuroprotection (Lendvai et al., 2011; Maison et al., 2013) and interaural balance (Darrow et al., 2006). Lesion studies showed that there was either no effect of lateral olivocochlear bundle lesions on hearing thresholds (Le Prell et al., 2003b; Darrow et al., 2007), or only a limited effect at high frequencies (Le Prell et al., 2005), but that evoked responses either decreased (Darrow et al., 2007) or increased (Le Prell et al., 2005) after removal of the lateral olivocochlear bundle feedback to the cochlea. These findings are not necessarily in opposition to our hypothesis, as we would rather predict an initial drop of spontaneous AN activity after a hearing loss with normalization over time (cf. below). Furthermore, and in support of our hypothesis it has been shown that the lateral olivocochlear bundle could provide the required noise input to the AN, as disruption of lateral olivocochlear neurons with a dopaminergic neurotoxin depressed spontaneous auditory nerve activity (Le Prell et al., 2014) and efferent synapses of the lateral olivocochlear bundle re-innervate IHCs of the aged cochlea (Lauer et al., 2012).

**Figure 9:**
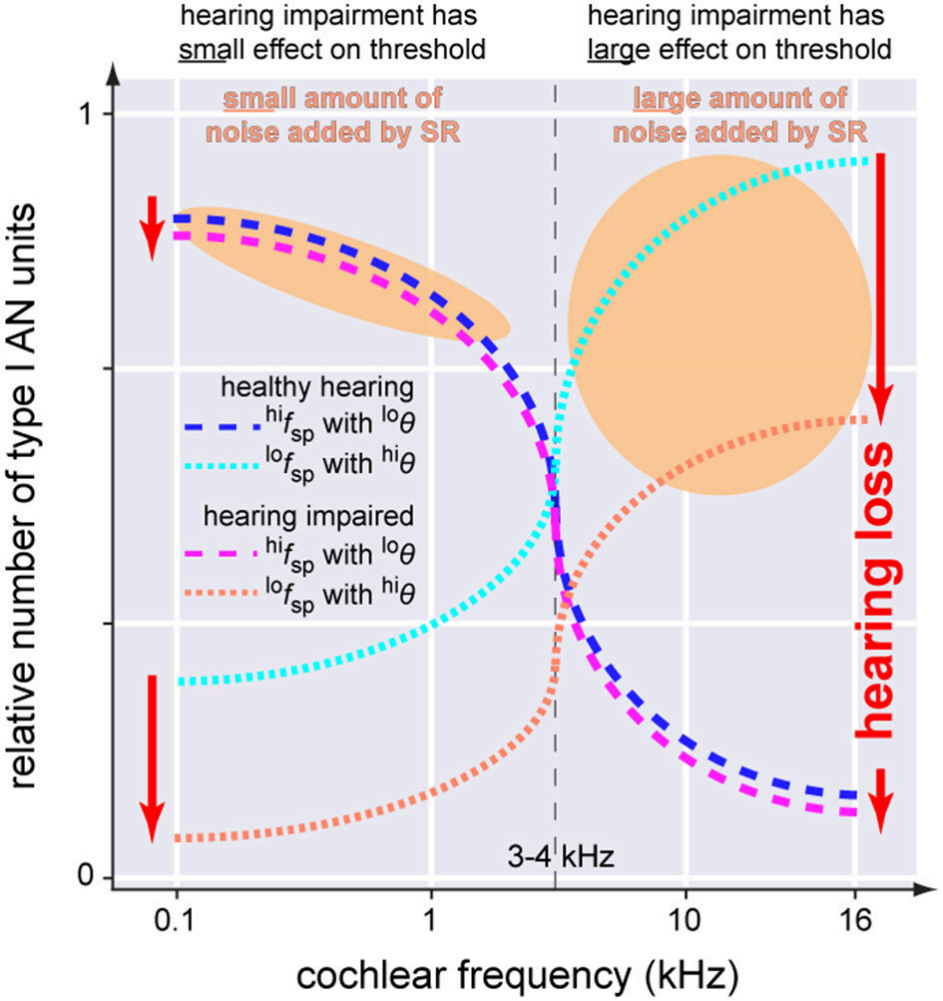
**Scheme of the anatomical asymmetry of threshold distributions of the AN type I units innervating the IHC**. In the healthy cochlea neurons with high spontaneous rates and low thresholds (blue dashed line, ^*hi*^*f*_*sp*_with ^*lo*^θ) are predominantly located below 3–4 kHz while units with low spontaneous rates and high thresholds (cyan dotted line, ^*lo*^*f*_*sp*_with ^*hi*^θ) are found across the whole cochlea. Following hearing impairment these latter neurons are affected first and most severely.(red dotted line) while the first fibers are mostly spared (magenta dashed line). SR increases noise intensity to counteract hearing loss (orange areas). Note that the noise intensity in the higher frequency range has to be much higher which may make it more likely for tinnitus to be induced in those frequencies ranges.

In this context it is worth mentioning that hearing loss—at least in rodents—has been found to predominantly cause loss of low spontaneous rate (^*lo*^*f*_*sp*_) AN fibers (Furman et al., 2013). In contrast to high spontaneous rate (^*hi*^*f*_*sp*_) fibers with a low threshold (^*lo*^θ), these fibers have comparatively high thresholds (^*hi*^θ) (Bourien et al., 2014). Interestingly, whereas ^*lo*^*f*_*sp*_ AN fibers are found across all frequency regions of the cochlea, ^*hi*^*f*_*sp*_ AN fibers are predominately found in the frequency regions of the cochlea below 3–4 kHz (Figure 9; Ohlemiller and Echteler, 1990; Heil and Peterson, 2015). Based on these findings it seems obvious that noise trauma would affect the high and low frequency region of the cochlea differently: as ^*lo*^*f*_*sp*_ AN fibers are more prone to get damaged by noise trauma than ^*hi*^*f*_*sp*_ AN fibers, the high frequency region would be more affected by the trauma than the low frequency region as there the more resilient ^*hi*^*f*_*sp*_ AN fibers are rare (cf. Figure 2 in Ohlemiller and Echteler, 1990). These findings are consistent with results from studies with human AN samples, describing a stronger loss of fibers in the basal compared to apical section of the cochlea (Zimmermann et al., 1995) in subjects with hidden and non-hidden hearing loss (Euteneuer and Praetorius, 2014). In the context of our model one may speculate that after trauma, SR may be more effective in the low frequency range below 3–4 kHz as there the number of ^*hi*^*f*_*sp*_ AN fibers is high (Figure 9). Furthermore, as they have ^*lo*^θ, for these fibers less internal noise would be needed to produce effective SR. By contrast, the few remaining ^*lo*^*f*_*sp*_ AN fibers in the high frequency range show ^*hi*^θ, so that much more internal noise would be needed for SR to be effective. This asymmetry of AN type I fiber innervation in the cochlea may therefore explain the asymmetry found in the patients' hearing threshold data. There, a threshold benefit was observed in tinnitus patients in the low frequency range only. If our model would be correct, this may further explain why tinnitus percepts are predominantly found in the high frequency range: there the system would aim to improve signal transmission by SR, but as thresholds of the remaining AN fibers are high, the high amount of internal noise added may be perceived as tinnitus which in turn could mask the possible threshold benefit introduced by SR, thereby finally resulting in overall elevated thresholds in tinnitus patients in that high frequency range (cf. below).

An alternative candidate structure for SR to be effective is the projection site of the AN at the dorsal cochlear nucleus (DCN): This hypothesis follows the basic idea that cochlear damage may result in reduced and therefore sub-threshold AN input to the DCN. This would be especially relevant in the context of light or hidden hearing loss, where the information from a diminished number of IHCs converge on DCN neurons and may not be sufficient to evoke a response there. In addition, the AN may also provide sub-threshold information of synchrony between different fibers (Young and Davis, 2002). As a source of noise background needed for SR—besides spontaneous activity generated within the DCN itself – also innervation from the somatosensory system seems conceivable (Dehmel et al., 2012) and may explain modulation of tinnitus sensation in patients, e.g., by jaw movements (Pinchoff et al., 1998).

In support of this view, in (animal) models of acoustic trauma induced tinnitus, increased spontaneous firing rates throughout the auditory system have been observed (Wang et al., 1997; Ahlf et al., 2012; Tziridis et al., 2015; Wu et al., 2016), and the DCN is the earliest processing stage in the auditory pathway in which acoustic trauma leads to tinnitus-related changes and increased spontaneous firing rates (Kaltenbach et al., 1998; Kaltenbach and Afman, 2000; Brozoski et al., 2002; Zacharek et al., 2002; Kaltenbach et al., 2004; Wu et al., 2016). The amount of this increase in spontaneous activity in the DCN has been shown to be correlated with the strength of the behavioral signs for tinnitus (Kaltenbach et al., 2004). Furthermore, this hyperactivity is only found in regions innervated by the damaged parts of the cochlear receptor epithelium (Kaltenbach et al., 2002) and is not brought back to normal levels even after cochlear ablation (Zacharek et al., 2002). Interestingly, Gao et al. recently described changes in DCN fusiform cell spontaneous activity after noise exposure that occur on short time scales (i.e., minutes). In contrast to HP mechanisms that work on long time scales (days or weeks), our SR mechanism may easily explain such fast adaptive dynamics reported by Gao et al. (2016). In particular and consistent with our model, the time course of spontaneous rate changes shows an almost complete loss of spontaneous activity immediately after loud sound exposure (as no SR is needed due to stimulation that is well above threshold), followed by an overcompensation of sound induced spontaneous rate changes to levels well above pre-exposition rates where SR is now needed to compensate for acute hearing loss (Gao et al., 2016).

Another argument in favor of the DCN as plausible site for SR relates to the information detector (Figure 1B) within our model which computes the autocorrelation of the AN activity. As has been pointed out in the Introduction section already, the evaluation of autocorrelation functions may easily be implemented within neuronal networks using delay-lines and coincidence detectors (Licklider, 1951). A neuronal architecture resembling such delay-lines has been described in the DCN (Osen, 1988; Hackney et al., 1990; Baizer et al., 2012).

Finally, as mentioned above, besides the possibility of noise generation within the DCN, the somatosensory projections to the DCN (Ryugo et al., 2003; Shore and Zhou, 2006; Dehmel et al., 2012; Zeng et al., 2012) could correspond to the noise generator (Figure 1B) of the model. Especially the integration over many different sensory systems within the DCN in combination with the shaping of these inputs by inhibition (Ryugo et al., 2003) may provide the ideal anatomical basis for noise propagation and adjustment: SR in this view would be controlled by inhibition of the noise generators within the DCN or of the noise fed back to the DCN from the somatosensory system, where a downregulation of inhibition (as a consequence of reduced input and therefore reduced autocorrelation) would increase internal noise by disinhibition of the noise generators or inputs.

We therefore believe that the DCN is the most likely structure within the auditory pathway where the sensor, the information detector and the noise adjusting structure of our model may be implemented. Beyond these speculations the most likely candidate structures for noise generation and propagation has to be the subject of future studies.

### Neuronal hyperactivity within the auditory system as a possible correlate for auditory phantom percepts

We have shown in this report that SR at the sensor level may be a mechanism to partly restore and thus improve information transmission into the auditory system after damage to the receptor epithelium, e.g., due to noise trauma. We have further demonstrated that for this mechanism to be effective, internal noise has to be generated, probably in form of increased spontaneous activity at some level within the auditory pathway. This may lead to higher sensitivity for even suprathreshold stimuli resulting in hyperacusis. This idea is in line with observations by Hébert and colleagues who have reported that hyperacusis, i.e., increased auditory sensitivity, is a pervasive complaint of people with tinnitus, suggesting that both symptoms have a common origin (Hébert et al., 2013).

In technical and physical systems the term *suprathreshold SR* has been coined for such a phenomenon (Collins et al., 1995; Stocks, 2000, 2001; Stocks; Stocks et al., 2002; McDonnell et al., 2008). Furthermore, we propose that in cases of permanently increased spontaneous activity, this hyperactivity would be able to induce neuronal plasticity along the auditory pathway that subsequently leads to the development of subjective, central tinnitus.

From human studies it is known that the presentation of external noise simultaneously to pure tones is actually able to significantly improve the hearing thresholds for these pure tones (Zeng et al., 2000; Long et al., 2004; Ries, 2007), a finding that was also explained by SR. Also our finding that across almost 40,000 patients those with tinnitus have lower hearing thresholds in the low frequency range than those without tinnitus has cursory been observed previously (König et al., 2006). In that study, König and colleagues described significantly better hearing thresholds for patients with tone-like tinnitus percepts compared to patients with noise-like percepts or without any tinnitus percept.

Our observation that tinnitus patients had increased hearing thresholds in the high frequency range is still in line with our model (cf. speculation about the loss of ^*lo*^*f*_*sp*_ *AN fibers*) but may need further inspection: As detailed above, the noise increase needed to produce effective SR in that high frequency range would have to be particularly large as thresholds there are disproportionately high and may therefore more likely induce tinnitus. In line with this rationale most tinnitus patients perceived their tonal tinnitus at high frequencies (Figure 7C). We therefore speculate that our finding of increased thresholds in high frequency ranges may result from masking of the hearing thresholds in the frequency range where tinnitus is perceived. The difference in mean audiogram observed in tinnitus patients compared to non-tinnitus patients may therefore be based on a combination of two effects: Primarily SR to restore hearing thresholds after hearing loss and subsequently masking of hearing thresholds by the SR-induced tinnitus. Whereas the former would be more effective in the low frequency range—due to the better survival of ^*hi*^*f*_*sp*_ fibers in the AN, the latter was more effective in the high frequency range. If this view would be correct, than the auditory system would improve impaired hearing thresholds in the frequency range relevant for speech processing at the cost of further impairing hearing thresholds in the high frequency range. Especially the effect of tinnitus pitch described by König et al. where high pitched tinnitus worsened hearing thresholds compared to low pitched tinnitus percepts (Figure 1C in König et al., 2006), supports our view of a masking effect in the high frequency range that counteracts the beneficial threshold shift based on SR.

In this context, one could interprete the patient data presented in Figure 7 as an extension of our single frequency channel model to a model with multiple independent frequency channels. The noise would be adjusted according to the local hearing threshold for the different frequency groups and not by a gain increase generalized to all frequency regions. This is consistent with the finding that in the DCN spontaneous activity has been shown to be correlated to the strength of the behavioral signs for tinnitus (Kaltenbach et al., 2004) and this hyperactivity is only found in regions innervated by the damaged parts of the cochlear receptor epithelium (Kaltenbach et al., 2002).

Another aspect that supports our hypothesis is the so called Zwicker tone illusion. The term describes an intriguing auditory aftereffect. The typical sound evoking a Zwicker tone is a broadband noise containing a spectral gap, which is presented for several seconds. After the sound has been switched off, a faint, almost pure tone is audible for 1 up to 6 s. It is decaying and has a sharp pitch in the spectral gap where no stimulus was available (Zwicker, 1964; Lummis and Guttman, 1972). Both the localization of the Zwicker tone in the brain and its origin has been long-standing open problems. In terms of our model we would speculate the cause of this auditory illusion to be the autocorrelation controlled upregulated internal noise for SR in response to the missing input within a certain spectral region introduced by the Zwicker paradigm. This is consistent with the suggestion that gain adaptation enhances internal noise of a frequency band otherwise silent due to damage (Parra and Pearlmutter, 2007).

Another interesting fact is that during the Zwicker tone sensation, auditory sensitivity for tone pulses at frequencies adjacent to the Zwicker tone are improved by up to 13 dB (Wiegrebe et al., 1996). It is plausible that cross-talk between adjacent frequency channels plays a role here. The sound intensities or AN firing rates of neighboring channels may serve as some kind of reference. Note that this threshold improvement again supports our hypothesis that SR plays a major role within the hearing system.

Furthermore, it is known that complete sensory deprivation may in some individuals lead to hallucination like experiences that occur after several hours in such a state (Lilly, 1956) and can produce acoustic phantom percepts as complex as music (Kjellgren et al., 2008). Other studies described tinnitus-like phenomena already after a few minutes in a sound proof chamber in 75% of the test subjects (Heller and Bergman, 1953). Finally, our model also easily explains why plugging of the outer ear canals also leads to perceptual changes like measurable improvement of hearing thresholds after unplugging or a transient tinnitus percept that vanishes after restoration of normal hearing (Schaette et al., 2012; Fournier et al., 2014).

In summary, we provide evidence that temporary and chronic auditory phantom percepts (Zwicker tones and tinnitus, respectively) may result from SR effects in the auditory pathway which have been evolutionary developed to counteract hearing loss. This alternative view opens up new perspectives for understanding the development of subjective tinnitus that will hopefully result in advanced therapeutic approaches to treat the condition. In this context, both adding external noise to induce SR, thereby superseding the internally generated neuronal noise, as well as strategies to suppress SR in the high frequency range with considerable tinnitus perception are conceivable.

## Author contributions

PK, AS, and CM performed the calculations for the models. PK, CM, KT, and HS discussed the theoretical background of the model. UH provided the human data. KT performed the analysis of the human data. PK, KT, and HS wrote the manuscript.

### Conflict of interest statement

The authors declare that the research was conducted in the absence of any commercial or financial relationships that could be construed as a potential conflict of interest.
